# Population pharmacokinetics of farletuzumab, a humanized monoclonal antibody against folate receptor alpha, in epithelial ovarian cancer

**DOI:** 10.1007/s00280-012-1959-y

**Published:** 2012-09-07

**Authors:** Colm Farrell, Charles Schweizer, Jason Wustner, Susan Weil, Masayuki Namiki, Tomohisa Nakano, Kenya Nakai, Martin D. Phillips

**Affiliations:** 1 ICON Clinical Research, Ellicott, MD USA; 2 Clinical Operations, Morphotek, Inc., 210 Welsh Pool Road, Exton, PA 19341 USA; 3 Eisai Co. Ltd., Tokyo, Japan

**Keywords:** Farletuzumab, Population pharmacokinetics, Monoclonal antibody, Ovarian neoplasms, Folate receptor alpha

## Abstract

**Purpose:**

The purpose of this analysis was to develop a population pharmacokinetic model for farletuzumab, a humanized immunoglobulin (Ig)G_1_ monoclonal antibody (mAb) to the folate receptor alpha, which is a receptor over-expressed in ovarian cancer, but largely absent from normal tissue.

**Methods:**

In total, 2,472 samples were included in the building of the pharmacokinetic model. Farletuzumab 12.5–400 mg/m^2^ had been administered via intravenous infusion to 79 patients with advanced ovarian cancer enrolled in one of the two clinical studies. Data were analyzed by a nonlinear mixed-effects modeling approach.

**Results:**

Farletuzumab pharmacokinetics was best described by a two-compartment model with first-order (linear) elimination. In the final model, estimated values of clearance and volume of distribution of the central compartment were 0.00784 l/h and 3.00 l, respectively. Body weight was the only covariate investigated that explained inter-patient variability in clearance and the central volume of distribution. There was no effect of age, human anti-human antibodies, or concomitant chemotherapy on the pharmacokinetics of farletuzumab. Simulations showed that, when the mg/kg/week dose was maintained, steady-state exposure to farletuzumab was similar with dosing every week or every 3 weeks.

**Conclusions:**

The pharmacokinetic parameters of farletuzumab are similar to those of other IgG mAbs. The results support weight-based dosing of farletuzumab on a weekly or 3-weekly schedule.

## Introduction

Ovarian cancer is the eighth most common cancer and fifth most common cause of cancer death in women in the United States [1]. The majority of women present with advanced disease, which is often highly sensitive to first-line chemotherapy treatment with platinum-based agents and taxanes following maximal cytoreductive surgery. However, most patients subsequently relapse and eventually die of disease persistence or recurrence resulting in low long-term survival rates. Five-year survival for women with ovarian cancer is approximately 45 % overall, but only 25 % in women with ovarian cancer that has metastasized [2]. Although the median survival of women with advanced disease is improving, there remains a pressing need for new approaches to enhance the management of these individuals.

One potential novel target is the folate receptor α (FRα). FRα is over-expressed in 90–100 % of epithelial ovarian cancers [3–5], but has limited expression in normal tissue. When FRα is expressed, it is restricted to the apical surfaces on polarized epithelial cells and is not exposed to the bloodstream [6, 7]. FRα is an interesting target, as the degree of FRα over-expression is correlated with both the stage and grade of disease and is a marker of more aggressive disease [3, 4]. In addition, over-expression of FRα enhances growth of tumorigenic cancer cells in vitro and in vivo [8].

Farletuzumab (MORAb-003) is a humanized immunoglobulin (Ig)G_1_ monoclonal antibody (mAb) directed against FRα and is in phase III development for ovarian cancer. In vitro, farletuzumab mediates complement-dependent and antibody-dependent cytotoxicity in tumor cell lines and inhibits FRα-dependent cell growth in CHO cells over-expressing the receptor [9]. In vivo, the murine LK26 FRα antibody (a precursor to farletuzumab) reduces tumor growth in a mouse xenograft model [9].

Data from a phase I clinical trial (NCT00428766) have shown farletuzumab to be generally well tolerated following intravenous (IV) administration and have provided an early indication of efficacy in patients with advanced ovarian cancer [10]. A small biodistribution sub-study using radiolabeled farletuzumab showed good tumor uptake of the mAb [11]. In a phase II trial (NCT00318370), farletuzumab in combination with platinum-based chemotherapy normalized levels of the tumor marker, CA-125, in approximately 90 % of women, and resulted in a second remission equal to, or longer than, the first remission in over 20 % of women [12]. Post hoc analysis showed the overall response rates to farletuzumab were similar in patients with first progression-free intervals of <12 and ≥12 months [12].

In the phase I and II studies, farletuzumab pharmacokinetics appeared to be dose-dependent [10, 12]. This paper reports a pooled population pharmacokinetic analysis based on data from the phase I and II studies, which aimed to characterize the pharmacokinetics of farletuzumab in patients with ovarian cancer, evaluate the effect of various potential covariates on the pharmacokinetics, and use simulations to support different dosing schedules.

## Materials and methods

### Study design and patients

The data used in this analysis were collected from two studies in women with advanced epithelial ovarian cancer conducted in accordance with Good Clinical Practice and under ethical principles established by the Declaration of Helsinki. All patients gave written informed consent.

The phase I study (NCT00428766; MORAb-003-001) was conducted in 25 patients with advanced epithelial ovarian, fallopian tube, or primary peritoneal cancer who had relapsed after failed standard chemotherapy. Farletuzumab was administered via IV infusion weekly for 4 weeks at ascending doses from 12.5 to 400 mg/m^2^ [10] to sequential cohorts of patients. The phase II study (NCT00318370; MORAb-003-002) was conducted in 54 patients with relapsed (asymptomatic or symptomatic) platinum-sensitive epithelial ovarian cancer, defined by elevated CA125 levels within 6–18 months of first remission. Patients received farletuzumab either as monotherapy (asymptomatic patients) or in combination with standard therapy (carboplatin and either paclitaxel or docetaxel). In combination with chemotherapy, farletuzumab was initially to be administered to six patients at each dose of 37.5 mg/m^2^ and 62.5 mg/m^2^; all remaining patients received farletuzumab at 100 mg/m^2^. Those responding to combination therapy continued on single-agent farletuzumab as weekly maintenance therapy. In both studies, infusions were commenced at 1 mg/min and advanced to 5 mg/min if tolerated.

### Blood sampling and analysis

In the phase I study, blood samples were taken pre-dose, mid-infusion, post–infusion, and at frequent intervals (30 min, and 1, 2, 4, and 24 h post-dose) for a period of 24 h post-dose (first and fourth dose) or 4 h (second and third dose). Additional sampling was conducted 2 weeks after the final dose. In the phase II study, blood samples were taken pre- and post-infusion weekly during cycle 1, then every 3 weeks. Additional samples were also taken 1 and 48 h post-infusion in cycles 1, 2, and 3 (monotherapy arm) or all cycles (combination therapy arm) from the first 13 enrolled patients participating in a pharmacokinetic sub-study.

Serum farletuzumab levels were assessed using a solid-phase capture enzyme-linked immunosorbent assay (ELISA) that used immobilized FRα to capture sample-based farletuzumab, followed by detection with a murine monoclonal anti-MORAb-003 IgG and an enzyme-conjugated secondary antibody. The low limit of quantitation was 3.13 ng/ml, equivalent to 313 μg/ml in undiluted serum.

### Population pharmacokinetic analysis

Pharmacokinetic data were analyzed using NONMEM program version VI and VII level 1.0 and 2.0, NM-TRAN version III level 1.0, and PREDPP version IV level 1.0 (ICON Development Solutions, Ellicott City, MD, USA). Pharmacokinetic parameters were estimated using first-order conditional estimation method with interaction (FOCEI). Only patients with evaluable dosing, actual sampling time, and farletuzumab concentration data were included in the analysis.

The population pharmacokinetic model was developed in a stepwise manner with evaluation at each step. Two initial pharmacokinetic models were assessed: a two-compartment model with either first-order elimination or with parallel Michaelis–Menten and first-order elimination. Model selection was based on goodness of fit plots, successful convergence, plausibility, and precision of parameter estimates, and minimum objective function value.

Distribution of inter-individual variability in the pharmacokinetic parameters was assumed to be log-normal and described by an exponential error model. Initial model building used a diagonal covariance matrix of inter-individual variability and a correlation between clearance and volume of the central compartment was included in the starting model. Residual error was modeled using combined additive and proportional models. Separate residual error terms were estimated for each of the two studies.

Covariate analyses were performed on clearance, volume of distribution of the central compartment and the peripheral compartment, and apparent inter-compartmental clearance. For covariates to be explored in the analysis, the covariate was required to have ≥10 % presence and sufficient range of values. Covariates included weight, body size (body mass index [BMI], body surface area [BSA]), age, human anti-human antibodies (HAHA), and concomitant chemotherapy.

Continuous covariates were entered into the model using the following equation:

$$ TVP_{i} = \, \theta_{1} * \, \left( {COV_{i} /COV_{ST} } \right)^{\theta } 2 $$

where *TVP*_*i*_ is the typical value of a pharmacokinetic parameter (*P*) for an individual (_*i*_), *COV*_*i*_ is the value of the covariate in the individual, *COV*_*ST*_ is the median value of the covariate in the study population, *θ*_*1*_ represents the typical value of the parameter, and *θ*_*2*_ represents the effect of the covariate on the parameter.

Categorical covariates were entered into the model using the following equation:

$$ TVP_{i} = \, \theta_{1} * \, \theta_{2}^{IND} i $$

where *TVP*_*i*_ is the typical value of a pharmacokinetic parameter (*P*) for an individual (_*i*_), *θ*_*1*_ represents the typical value of the parameter in the absence of the covariate (when *IND*, the indicator variable, is equal to zero), and *θ*_*2*_ is the fractional change in the typical value if the covariate is present (*IND* = 1).

Covariates were modeled individually. Only those found to influence pharmacokinetic parameters were included in the final model. Significance was confirmed using a backward elimination procedure.

The effective half-life (t_1/2,eff_) was calculated according to the following equation:

$$ {\text{t}}_{{ 1/ 2,{\text{eff}}}} = { \ln }\left( 2\right)/{\text{K}}_{\text{eff}} {\text{where K}}_{\text{eff}} = \, - { \ln }\left( { 1- \left( {{\text{AUC}}_{(0-\tau )} /{\text{AUC}}_{{{\text{ss}},\tau }} } \right)} \right)/\tau $$

where K_eff_ is an effective rate constant, AUC_(0–τ)_ is the area under the concentration time curve from 0 to τ hours following the first dose, AUC_ss,τ_ is the AUC over a steady-state dosing interval, and τ is the dosing interval.

### Model evaluation

The final farletuzumab population pharmacokinetics model was used to simulate 250 replications of the observed dataset. The observed data were compared with the fifth, tenth, ninetieth, and ninety-fifth percentile of the simulated data. The model was evaluated with a visual predictive check, and the numbers of observed concentrations falling within 80 and 90 % prediction intervals of the simulated data were determined.

## Results

### Patient population

The final pharmacokinetic dataset included 2,472 samples from 79 women (Table 1).

**Table 1:** Patient demographics

	Phase I study MORAb-003-001	Phase II study MORAb-003-002	Overall
No. of patients	25	54	79
Weight (kg), median (range)	67.5 (44.5–87.0)	66.1 (44.5–118.2)	66.2 (44.5–118.2)
Age (year), median (range)	56 (44–79)	64 (31–81)	61 (31–81)
BMI (kg/m^2^), median (range)	25.4 (17.4–35.2)	27.3 (19.3–47.9)	26.5 (17.4–47.9)
BSA (m^2^), median (range)	1.67 (1.44–2.02)	1.69 (1.37–2.24)	1.69 (1.37–2.24)
Race			
Caucasian	17	44	61
African–American	1	1	2
Asian	1	5	6
Hispanic	1	4	5
Other	5	0	5

*BMI* body mass index, *BSA* body surface area

Women were aged between 31 and 81 years with a mean weight of 66.2 kg. In the phase I study, 15/25 women had a Karnofsky performance status value of 90 % (10 with Karnofsky status of 80 %), and in the phase II study, 36/54 had an ECOG performance status of 0 (18 with ECOG status 1). All patients in the phase I study received farletuzumab monotherapy. In the phase II study, 28 patients initially started on farletuzumab monotherapy; 21/28 went on to receive combination therapy and then 16/21 continued with farletuzumab monotherapy. Twenty-six patients started on combination therapy and 20/26 continued with farletuzumab monotherapy. Thus, over the course of the two studies, farletuzumab monotherapy was received by 73 women, and farletuzumab in combination with standard chemotherapy was received by 47 women.

On average, more than 30 samples were available per patient. More than half of the samples in the analysis were obtained from women receiving farletuzumab 100 mg/m^2^ (Table 2).

**Table 2:** Pharmacokinetic dataset

Dose (mg/m^2^)	Phase I study		Phase II study		Overall	
	No. of patients	No. of samples	No. of patients	No. of samples	No. of patients	No. of samples
12.5	3	81	–	–	3	81
25.0	3	88	–	–	3	88
37.5	3	89	4	211	7	300
62.5	3	90	5	250	8	340
100.0	3	91	45	1,289	48	1,380
200.0	3	95	–	–	3	95
400.0	7	188	–	–	7	188
Total	25	722	54	1,750	79	2,472

### Population pharmacokinetic model

Combined data from both studies were best described by a two-compartment model with first-order (linear, dose-independent) elimination rather than a two-compartment model with parallel Michaelis–Menten and first-order elimination. There was some evidence of nonlinearity at very low doses (namely, dose-dependency in clearance with farletuzumab 12.5 and 25.0 mg/m^2^); however, analysis of full datasets from both studies, with a two-compartment model with parallel Michaelis–Menten and first-order elimination, did not support nonlinearity. Therefore, the 169 observations at low doses (12.5 and 25.0 mg/m^2^) were excluded for final model development and the two-compartment model with first-order elimination applied to the revised dataset.

In the base model, pharmacokinetic parameter estimates for farletuzumab showed slow clearance (0.00830 l/h) and small distribution volumes (3.00 l and 6.51 l for the volume of distribution of the central and peripheral compartments, respectively). The inter-compartmental clearance was 0.0213 l/h. The inter-individual variability for both clearance and volume of distribution of the central compartment was <35 %, although inter-individual variability of the peripheral volume of distribution was higher (103 %).

The potential influence of several demographic/covariate factors on the pharmacokinetics of farletuzumab was explored. Body weight had the largest effect on clearance and volume of distribution of the central compartment, but had no effect on peripheral volume of distribution or inter-compartmental clearance. Inclusion of BSA or BMI into the model did not improve the accuracy of the model to predict pharmacokinetic parameters. There was no significant effect of age on any of the pharmacokinetic parameters investigated. Additionally, a review of the available HAHA data indicated that infrequent low-level HAHA formation in study patients had no significant impact on farletuzumab exposure. The effect of concomitant chemotherapy on the pharmacokinetic parameters of farletuzumab was investigated last and was found to have no statistically or clinically significant effects on the parameters tested.

The pharmacokinetic parameter estimates of the final population model are shown in Table 3. Clearance was estimated at 0.00784 l/h, and the volumes of distribution of the central and peripheral compartments were estimated at 3.00 and 7.50 l, respectively. Inter-compartmental clearance was estimated at 0.0203 l/h.

**Table 3:** Parameter estimates for the final population pharmacokinetic model

	Estimate	% RSE	95 % CI	
Parameter				
CL (l/h)	0.00784	5.79	0.00695, 0.00873	
Vc (l)	3.00	5.20	2.69, 3.31	
Q (l/h)	0.0203	4.46	0.0185, 0.0221	
Vp (l)	7.50	20.30	4.52, 10.50	
Covariate influence				
CL ~ WT	0.715	35.2	0.221, 1.210	
Vc ~ WT	0.629	30.2	0.257, 1.000	
Inter-individual variability				% CV
ω^2^ CL	0.0616	27.8	0.0281, 0.0951	24.8
ω^2^ Vc	0.0470	33.6	0.0160, 0.0780	21.7
ω^2^ Vp	1.180	21.3	0.688, 1.670	109.0
Residual variability				% CV or SD
Phase I study				
σ^2^ proportional (% CV)	0.0420	5.07	0.0378, 0.0462	20.5
σ^2^ additive (SD μg/ml)	0 (fixed)	–	–	–
Phase II study				
σ^2^ proportional (% CV)	0.122	1.87	0.118, 0.126	34.9
σ^2^ additive (SD μg/ml)	63.0	4.71	57.2, 68.8	7.94

*%RSE* percent relative standard error of the estimate, *ω* ^*2*^ variance of the inter-individual random effect, *σ* ^*2*^ variance of the residual intra-individual random error, *CI* confidence interval, *CL* clearance, *CV* coefficient of variation, *Q* apparent inter-compartmental clearance, *SD* standard deviation, *Vc* volume of distribution of the central compartment, *Vp* volume of distribution of the peripheral compartment, *WT* weight

Weight had a greater influence on the volume of distribution of the central compartment than on clearance: estimates of the exponents of weight-based allometric scaling were 0.715 and 0.629, respectively, and relative standard errors of these estimates were <36 %. With the inclusion of weight as a covariate, the inter-individual variability of clearance was reduced from 34.4 to 24.8 %, and the inter-individual variability of the central volume of distribution was reduced from 25.6 to 21.7 %.

The residual variability estimated for the phase II study was higher than that for the phase I study (34.9 and 20.5 %, respectively).

### Model evaluation

**Fig. 1:**
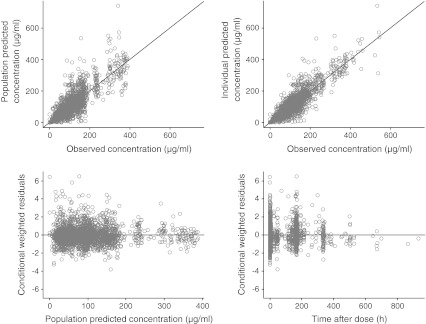
Farletuzumab final pharmacokinetic model diagnostic plots of observed versus predicted concentrations and conditional residuals versus predicted concentrations

The diagnostic plots of predicted and observed data (Fig. 1) indicated that the model described the observed plasma farletuzumab concentration data well. Re-introduction of the 169 observations associated with farletuzumab 12.5 and 25.0 mg/m^2^ had little impact on clearance and the central volume of distribution estimates, but increased inter-individual variability and resulted in the effect of body weight on these parameters being less well estimated.

**Fig. 2:**
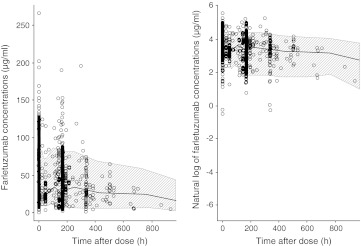
Visual predictive check for the final farletuzumab pharmacokinetic model

The visual predictive check of the final pharmacokinetic model confirmed the suitability of the model (Fig. 2). Only 9.37 % of observations fell outside the 90 % prediction interval.

### Half-life calculations

Using the individual pharmacokinetic parameter estimates from the final model, the alpha (distribution) and beta (elimination) half-lives of farletuzumab were calculated to be 2.48 (95 % confidence interval [CI] 1.00, 3.38) days and 49.2 (95 % CI 14.6, 173.0) days, respectively. Based on an accumulation ratio of 3.11 following farletuzumab dosing once weekly, the effective or functional half-life of farletuzumab was calculated to be approximately 12.5 days.

## Discussion

This is the first population pharmacokinetic model developed for farletuzumab using combined data from a phase I and a phase II study. Farletuzumab pharmacokinetics were best described by a two-compartment model with linear elimination over a dose range of 37.5–400 mg/m^2^, although there was some evidence of nonlinear pharmacokinetics at very low farletuzumab doses (12.5 and 25.0 mg/m^2^). A two-compartment model has been used previously to describe the pharmacokinetic data of all intravenously administered monoclonal antibodies, many (e.g., bevacizumab, pertuzumab, and trastuzumab) with linear first-order clearance, independent of dose [13–15]. The nonlinearity at very low doses has also been observed previously with bevacizumab [14], where clearance was 2–3 times faster with doses of 0.1 and 0.3 mg/kg than at doses ≥1 mg/kg. As the farletuzumab 12.5 and 25.0 mg/m^2^ doses are considerably lower than doses proposed for further clinical study, it was considered appropriate to exclude them from the final model.

Estimates showed farletuzumab had a small volume of distribution (indicating limited tissue penetration) and slow clearance (0.188 l/day) comparable to that of other monoclonal antibodies (0.207 l/day for bevacizumab, 0.214 l/day for pertuzumab, and 0.225 l/day for trastuzumab) [13–15]. The large molecular weight of mAbs and their hydrophilicity/polarity explain the consistently small volumes of distribution of farletuzumab and other mAbs. Central and peripheral volumes of distribution are in the range 2.4–5.5 l and 1.3–7.5 l, respectively [16].

Covariates of size (weight, BMI, and BSA) are the most frequently identified and clinically relevant covariates of the pharmacokinetics of mAbs [16]. In this analysis, weight was found to influence both farletuzumab clearance and central volume of distribution. Inclusion of weight within the model reduced the inter-individual variability of clearance and central volume of distribution, but inclusion of either BSA or BMI within the model did not improve its accuracy. This is consistent with what was expected on the basis of other population pharmacokinetic analyses where weight is most commonly included in the final model [16]. Accordingly, and aligned with the approved dosing of approved mAbs, ongoing studies of farletuzumab in ovarian cancer and other tumor types are using weight-based, mg/kg, dosing.

The other covariates investigated, including age, did not affect farletuzumab pharmacokinetic parameters in this model. In other analyses, age has only been identified as a covariate in the population pharmacokinetic analysis of efalizumab [17] (which was attributed to the subcutaneous route of administration of efalizumab and the aging characteristics of the skin) and panitumumab [18].

Concomitant chemotherapy also did not affect farletuzumab pharmacokinetic parameters. Because mAbs are not substrates for the enzyme systems, such as cytochrome P450, involved in the metabolism of small molecules, pharmacokinetic drug–drug interactions between mAbs and small-molecule chemotherapy are not anticipated [19]. Indeed, these have not been shown with other mAbs other than with a couple of exceptions [16, 19], although the ongoing potential for drug–drug interactions is being investigated in studies with farletuzumab where combination therapy is being given.

As mAbs are exogenous proteins, they can elicit an immune response with endogenous antiglobulins targeting the mAb. Despite the assay method used to detect HAHA to farletuzumab being extremely sensitive (lower limit of detection of 8 ng/ml), HAHA incidence level was very low, and the vast majority of the positive values were threshold responses, close to the assay cut-off point in both the phase I and phase II studies [10, 12]. Given the low magnitude in all but a few patients, these immune responses were unlikely to alter farletuzumab pharmacokinetics significantly.

Although formation of HAHA and binding of these to farletuzumab could alter elimination rates (as the resulting immune complexes are cleared more quickly than IgG_1_ antibodies), a review of the available HAHA data indicated no clear impact on farletuzumab exposure. It should be noted, however, that the presence of elevated levels of free unbound drug in serum samples may confound the detection and impact of HAHA, in particular against farletuzumab, as method validation demonstrated drug tolerance of approximately 100 ng/ml. Nevertheless, the low level of HAHA formation coupled with high circulating farletuzumab concentrations makes it unlikely that any immunocomplexes significantly altered the pharmacokinetic evaluation.

Gender and race were not investigated as covariates, as all patients were female and the majority were Caucasian. Although gender has been identified as a predictor of clearance and the volume of distribution of the central compartment with a minority of mAbs, including bevacizumab [14], the magnitude of the effect is not sufficient to require dose adjustments in males compared with females. Although all completed studies with farletuzumab thus far have involved women, current investigations in other tumor types involve patients of both genders. Race has not been found to influence the pharmacokinetics of many mAbs [16]. Similarly, as expected from the limited involvement of the kidney and liver in the clearance of mAbs, hepatic and renal functions have rarely been identified as covariates in population pharmacokinetic analyses of mAbs [16, 20] and were not investigated as covariates in the farletuzumab model.

Residual variability was modeled using combined proportional and additive components for each study. The proportional residual variability associated with the phase II study was higher than that for the phase I study, but both were consistent with the proportional residual variability of other MAb pharmacokinetic models (8.8–42.0 % [16]). The lower residual variability in the phase I study is not unexpected given that timings associated with drug administration and blood sampling tend to be managed particularly stringently in phase I studies.

Using elimination half-life as an indication of dosing frequency is scientifically reasonable for most drugs with rapid absorption. However, elimination half-life alone cannot be used to guide dosing frequency for drugs with extended absorption or for drugs with multiphasic disposition where the contribution of the terminal phase to steady-state exposure is limited. In such cases, an ‘effective’ or ‘functional’ half-life pharmacokinetic parameter better guides dosing frequency [21]. In this analysis, the effective half-life of farletuzumab (estimated at 12.5 days) was much shorter than the elimination half-life, suggesting the elimination half-life makes a relatively small contribution to the accumulation of farletuzumab when administered once weekly. Dose simulation studies (Eisai, data on file) predicted that farletuzumab accumulation over a 3-week period is similar with 3-weekly dosing compared with once-weekly dosing, when farletuzumab is dosed at 3 times the weekly dose. Within the ongoing clinical development program for farletuzumab, a variety of different dosing schedules are being employed.

In summary, farletuzumab accumulation to steady state can be predicted by a linear two-compartment pharmacokinetic model. The pharmacokinetic parameters of farletuzumab and observed variability of these parameters are typical of other IgG mAbs. Identification of weight as the covariate with the greatest influence of farletuzumab pharmacokinetics supports dosing on a mg/kg basis. Similar exposure to farletuzumab can be achieved independently of dosing schedule, with the results supporting dosing of farletuzumab every 1, 2, or 3 weeks.
